# Characterisation of the canine faecal virome in healthy dogs and dogs with acute diarrhoea using shotgun metagenomics

**DOI:** 10.1371/journal.pone.0178433

**Published:** 2017-06-01

**Authors:** Paloma S. Moreno, Josef Wagner, Caroline S. Mansfield, Matthew Stevens, James R. Gilkerson, Carl D. Kirkwood

**Affiliations:** 1 Enteric Viruses Group, Murdoch Childrens Research Institute, Melbourne, Victoria, Australia; 2 Translational Research and Animal Clinical Trial Study (TRACTS) group, Faculty of Veterinary and Agricultural Sciences, The University of Melbourne, Melbourne, Victoria, Australia; 3 Department of Paediatrics, The University of Melbourne, Melbourne, Victoria, Australia; 4 Australian Genome Research Facility, Melbourne, Victoria, Australia; 5 Centre for Equine Infectious Diseases, Faculty of Veterinary and Agricultural Sciences, The University of Melbourne, Melbourne, Victoria, Australia; Universitatsmedizin Greifswald, GERMANY

## Abstract

The virome has been increasingly investigated in numerous animal species and in different sites of the body, facilitating the identification and discovery of a variety of viruses. In spite of this, the faecal virome of healthy dogs has not been investigated. In this study we describe the faecal virome of healthy dogs and dogs with acute diarrhoea in Australia, using a shotgun metagenomic approach. Viral sequences from a range of different virus families, including both RNA and DNA families, and known pathogens implicated in enteric disease were documented. Twelve viral families were identified, of which four were bacteriophages. Eight eukaryotic viral families were detected: *Astroviridae*, *Coronaviridae*, *Reoviridae*, *Picornaviridae*, *Caliciviridae*, *Parvoviridae*, *Adenoviridae* and *Papillomaviridae*. Families *Astroviridae*, *Picornaviridae* and *Caliciviridae* were found only in dogs with acute diarrhoea, with *Astroviridae* being the most common family identified in this group. Due to its prevalence, characterisation the complete genome of a canine astrovirus was performed. These studies indicate that metagenomic analyses are useful for the investigation of viral populations in the faeces of dogs. Further studies to elucidate the epidemiological and biological relevance of these findings are warranted.

## Introduction

Interest in the virome, or the entire population of viruses present in a biological sample, has increased recently due to improved availability of high throughput sequencing or next generation sequencing (NGS) technologies, and improved metagenomic analytical methods [1, 2]. The virome comprises all types of viruses, including those that infect prokaryotic and eukaryotic organisms, DNA or RNA viruses, and viruses that cause acute or chronic infections. Many of these viruses are difficult or impossible to propagate in cell culture, and molecular detection is difficult as no common gene such as the ribosomal 16S gene that is present in bacterial species exists in viruses. These limitations have hindered the identification and characterisation of uncultured viruses [3, 4]. Recently, due to the advent of molecular enrichment protocols, high throughput sequencing and new metagenomic analytical methods we are now able to explore, identify and characterise viruses from different biological and environmental samples with a greater capacity [2, 5–11]

In studies of human faeces, the virome has been shown to include viruses that infect eukaryotic organisms and viruses that infect prokaryotes (bacteriophages) [2, 5, 12–18]. Bacteriophages have been reported in many studies to be the most frequently detected viral constituent in the gut of humans [1, 2, 5, 8, 16, 19, 20]. The faecal virome has been characterised for several animal species including pigs, bats, cats, pigeons, horses and ferrets [2, 6, 7, 9–11, 21–31]. In dogs, the presence of enteric viral pathogens such as canine parvovirus, coronavirus, rotavirus and distemper virus (*Paramyxoviridae*) have been identified only through targeted studies [32–35]. To date, only one published study has used high throughput sequencing to investigate the faecal viral population in diarrhoeic dogs [6]. These investigators analysed faeces from dogs with acute diarrhoea and detected two new virus species, canine sapovirus and canine kobuvirus; known canine enteric viruses such as canine coronavirus, canine parvovirus, canine rotavirus as well as plant and insect viruses were also reported [6].

The aim of this study was to describe the faecal virome of samples collected from healthy dogs, and compare these findings to the faecal virome of dogs with acute diarrhoea in Australia, using an Illumina MiSeq shotgun metagenomic sequencing approach.

## Results

### Overview of the canine faecal virome

**Fig 1:**
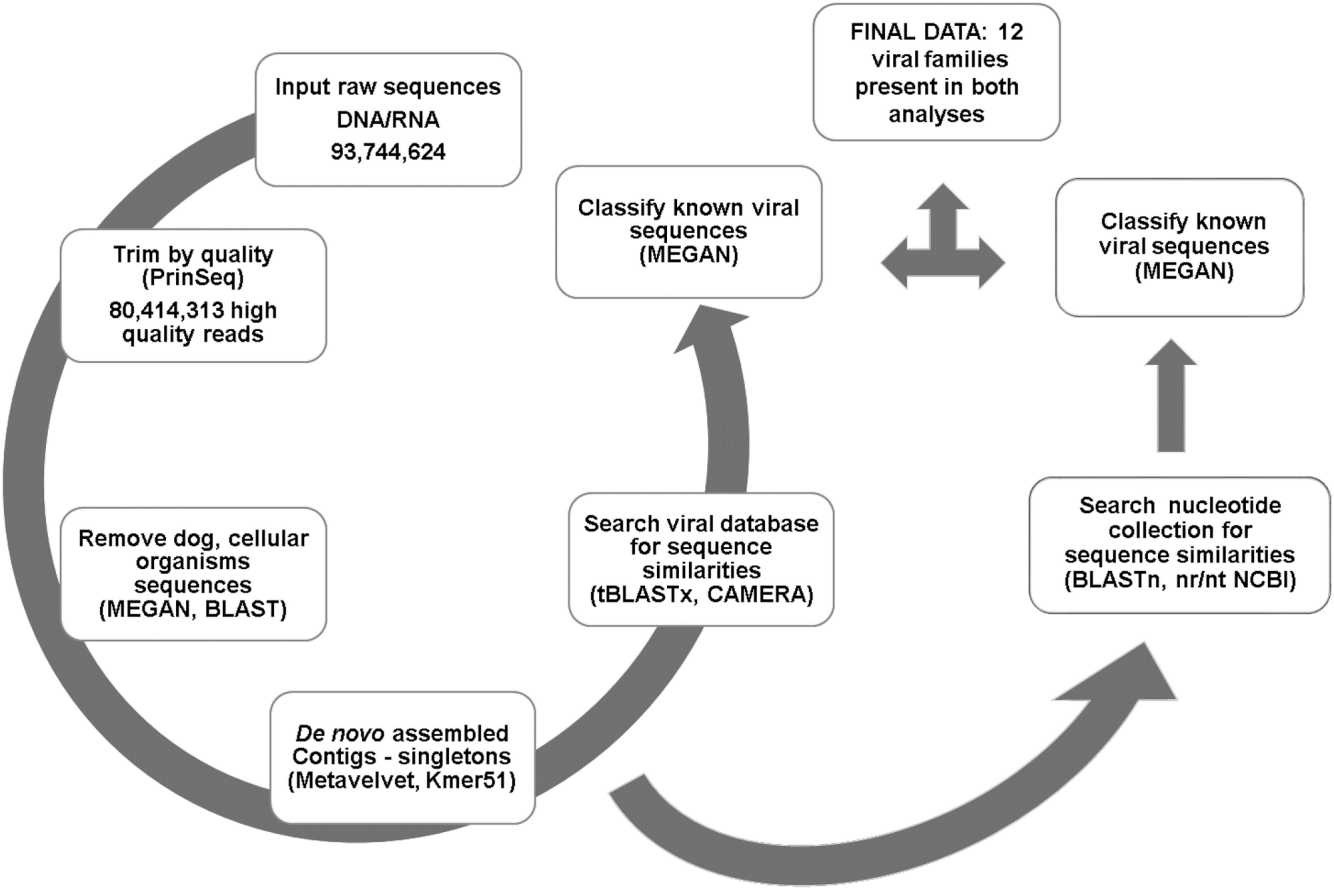
Summary of the bioinformatic pipelines after sequencing, showing results obtained in some steps.

A total of 16 faecal samples (8 from healthy and 8 from diarrhoeic dogs) were subjected to viral nucleic acid extraction, followed by nucleic acid enrichment, reverse transcription, random amplification and the creation of two libraries for each sample (DNA and cDNA), before being sequenced by Illumina MiSeq platform (Table 1). After sequencing, a total of 93,744,624 raw sequences were generated. All raw sequences are available in NCBI, (Bioproject ID: PRJNA380672). After trimming by quality 80,414,313 high quality reads (HQRs) were available. All sequences corresponding to dog and cellular organisms (383,785 and 27,825,631 respectively) were removed and the resultant reads were *de novo* assembled (Fig 1) generating in total 1,672,615 contigs and singletons (reads). From these contigs/singletons 1,285,171 (76.8%) had no hits in the database (S1 Table). Further analysis of contigs/singletons with no hits confirmed most sequences had no hits, while a limited number matched bacterial, human or animal sequences with a very low coverage. In addition to the contigs/singletons with no hit in the database, some contigs/singletons matching to cellular organisms and some with low complexity were identified, however, were not analysed any further (S1 Fig).

**Table 1:** Summary of clinical information and contigs/singletons of eukaryotic viral families detected by metagenomic sequencing in faeces of dogs.

SAMPLE	SEX	BREED	AGE	TOTAL N° PROKARYOTICCONTIGS/SINGLETONS	EUKARYOTIC VIRAL FAMILIES (N° OF CONTIGS/SINGLETONS DETECTED)	LOWEST COMMON ANCESTOR ACCORDING TO MEGAN (N° OF CONTIGS/SINGLETONS DETECTED)
**ND4**	Male Neutered	Maltese cross	7 years	1012	*Reoviridae* (39)	Rotavirus A (22)
**ND5**	Male Neutered	Jack Russell	2 years	209	None	None
**ND6**	Female	Maltese/Shih Tzu	5 years	953	None	None
**ND7**	Female Neutered	Staffordshire	4 years, 7months	731	None	None
**ND8**	Male Neutered	Labrador cross	7 years	82	None	None
**ND9**	Male Neutered	Mastiff cross	8 months, 1week	5	*Parvoviridae* (3)	Canine parvovirus (3)
**ND10**	Male	Maltese cross	8 months	2	*Coronaviridae* (912)	Alphacoronavirus 1, FIPV (76)
**ND11**	Male Neutered	Maltese/Shih Tzu	2 years, 6months	18	*Adenoviridae* (1)	Human adenovirus C (1)
					*Papillomaviridae* (1)	Human papillomavirus type 118 (1)
**DD1**	Male	Mastiff cross	2 years	1777	*Astroviridae* (18)	Canine astrovirus (5)
					*Picornaviridae* (3)	Canine kobuvirus (3)
**DD2**	Male	Mastiff cross	4 months	2179	*Parvoviridae* (41)	Canine parvovirus (41)
					*Astroviridae* (3)	Canine astrovirus (3)
**DD3**	Female Neutered	Wirehair Dachshund	5 years	1559	*Reoviridae* (2)	Rotavirus A (2)
					*Astroviridae* (3)	Canine astrovirus (3)
					*Coronaviridae* (4)	Alphacoronavirus 1, FIPV (3)
**DD5**	Male	Husky	10 weeks	1387	*Astroviridae* (3)	Canine astrovirus (3)
					*Caliciviridae* (8)	Canine norovirus (6)
**DD7**	Male	Husky	10 months	2593	*Reoviridae* (3)	Rotavirus A (3)
**DD8**	Female Neutered	German shorthair pointer	7 months	3230	*Parvoviridae* (3)	Canine parvovirus (3)
					*Coronaviridae* (211)	Alphacoronavirus 1, FIPV (36)
**DD9**	Female	Bull dog	4 years	3319	*Reoviridae* (5)	Rotavirus A (1)
**DD10**	Male	Rhodesian ridgeback	2 years	886	*Reoviridae* (4)	Rotavirus A (4)
					*Astroviridae* (1)	Canine astrovirus (1)

**ND**: normal dogs, **DD**: dogs with acute diarrhoea.

Sequences similar to twelve viral families were identified in faecal samples from healthy and diarrhoeic dogs after analyses with two different bioinformatic pipelines and comparison against viral and NCBI databases (Fig 1). Eight of these viral families infect eukaryotic organisms, and the remaining four infect prokaryotes.

Despite the known bias of SISPA in the resultant sequences after *de novo* assembly [36], we report the number of contigs/singletons matching viral families and the subsequent analysis of alignments with the lowest common ancestor according to MEGAN V5.2.1 [37].

### Virome of healthy dogs

Faecal samples were collected from eight healthy dogs (Table 1). Genetic analyses identified 659,696 contigs/singletons with no hits and 3968 contigs/singletons were classified as viral, matching to five viral families that infect eukaryotes and four that infect prokaryotes. 75.9% (3012 contigs/singletons) of the total number of viral contigs/singletons were classified as bacteriophages in the healthy canine faecal virome. Bacteriophages were detected in the faeces of all dogs in this group and belonged to *Caudovirales* order and *Microviridae* family.

Viral contigs/singletons from five eukaryotic virus families were identified in faecal samples from 4 of the 8 healthy dogs (Table 1). Three out of five viral families detected were DNA viruses. *Adenoviridae* and *Papillomaviridae* were detected in a single sample containing only one contig/singleton each. The contig corresponding to *Papillomaviridae* family aligned with human papillomavirus type 118 (GQ246951.1), and covered 1.7% of complete genome (minimum match: 70% and minimum overlap: 30). A similar situation was identified for the Adenovirus contig. Genetic analysis revealed it matched human adenovirus C (NC_001405.1) and only covered 0.34% of the genome. This healthy individual dog sample (ND11) was the only one that had co-infection with different eukaryotic virus families in this group.

The highest number of contigs/singleton detected (n = 912) were from the family *Coronaviridae* (Table 1), however, these were all detected in one sample. After analysis, only 76 contigs/singletons matched the reference sequence of Alphacoronavirus 1 (Feline infectious peritonitis virus, NC_002306.3) and covered only 0.5% of the complete genome (minimum match: 75% and minimum overlap: 50), which represented 3.2% of FIPV_gp02 (receptor binding molecule) region.

Contigs/singletons belonging to the *Reoviridae* family were found in only one sample. Genetic analysis revealed they covered between 11%–35.5% of VP1, VP2, VP3 and VP4 genes of reference sequences of Rotavirus A (NC_011506–NC_011510).

Another eukaryotic viral family found in one healthy dog sample was *Parvoviridae*, genetic analysis of the 3 contigs/singletons showed a coverage of approximately 3.5% of the complete genome of canine parvovirus reference sequence (NC_001539), or 9.3% of the polyprotetin Ns1-Ns2.

### Virome of dogs with acute diarrhoea

In eight faecal samples from dogs with acute diarrhoea, a total of 625,475 contigs/singletons had no hits and 17,242 were identified as viral contigs/singletons comprising 6 eukaryotic and 4 prokaryotic viral families (Table 1). Bacteriophages comprised 98.19% of the total of viral contigs/singletons and they were present in all individuals and were identified as belonging to order *Caudovirales* and *Microviridae* family. Eukaryotic families found in this group were *Coronaviridae*, *Parvoviridae*, *Reoviridae*, *Caliciviridae*, *Astroviridae*, and *Picornaviridae* (Table 1). The most common eukaryotic viruses identified were RNA viruses (5/6 viral families). Interestingly, all 8 samples in this group contained at least one eukaryotic family each. Co-infection was identified in 6 individual dog samples from this group. From the 8 samples from dogs with acute diarrhoea, 2 different eukaryotic virus families were detected in five samples and 3 eukaryotic families were detected in one sample (Table 1). The most prevalent family identified was *Astroviridae*, present in 5 dogs followed by *Reoviridae* present in 4 of 8 dogs with acute diarrhoea (Table 1).

*Astroviridae* contigs/singletons from 5 dogs were compared with reference sequence of canine astrovirus (NC_026814.1), the lowest common ancestor according to MEGAN, and they covered between 2.4% and 5% of compete genome and between 6.3% and 12.9% of the ORF2. The sample with the most contigs/singletons was later characterised.

*Reoviridae* contigs/singletons found in 4 dogs were compared with reference sequence of Rotavirus A (NC_011503.2) and 3 of them covered between 10.5% and 23.4% of the VP4 gene and the other sample covered 23.6% of the VP7 gene.

Furthermore, contigs/singletons matching to canine parvovirus, were found in 2 dog samples with acute diarrhoea. One of the samples covered approximately 5.8% of the complete genome of canine parvovirus reference sequence (NC_001539), corresponding to 17.6% of VP2. The other sample contained 41 contigs/singletons that matched to this same reference sequence and in total they cover 100% of VP1, 66.2% of polyprotein NS1 and NS2 (CPVgp1) and 87.9% of VP2 genes.

In this group were also found contigs/singletons matching to the *Coronaviridae* family, covering between 0.6% and 1.7% of the complete genome of reference sequence Alphacoronavirus 1 (FIPV, NC_002306.3).

One dog with acute diarrhoea contained contigs/singletons similar to a canine norovirus (JF930689.1), covering approximately 7.9% of the complete genome. Other dog sample had contigs/singletons similar to a canine kobuvirus (JN387133.1), covering 2.2% of complete genome.

### Canine astrovirus characterisation

To further explore the high abundance of contigs/singletons from *Astroviridae* family in dogs with acute diarrhoea and their absence in healthy dogs, a near complete full genome of a representative canine astrovirus was generated through Sanger sequencing (DD1, Table 1). The genome encoded the complete three open reading frames (ORFs): ORF1a, ORF1b and ORF2. The total length was 6513 nucleotides, excluding the 3’ poly (A) tail and the nucleotide composition was 28% A, 22% G, 26% T, 23% C. The G/C composition was 45%. The GenBank accession number for the canine astrovirus sequence is KX756441.

**Fig 2:**
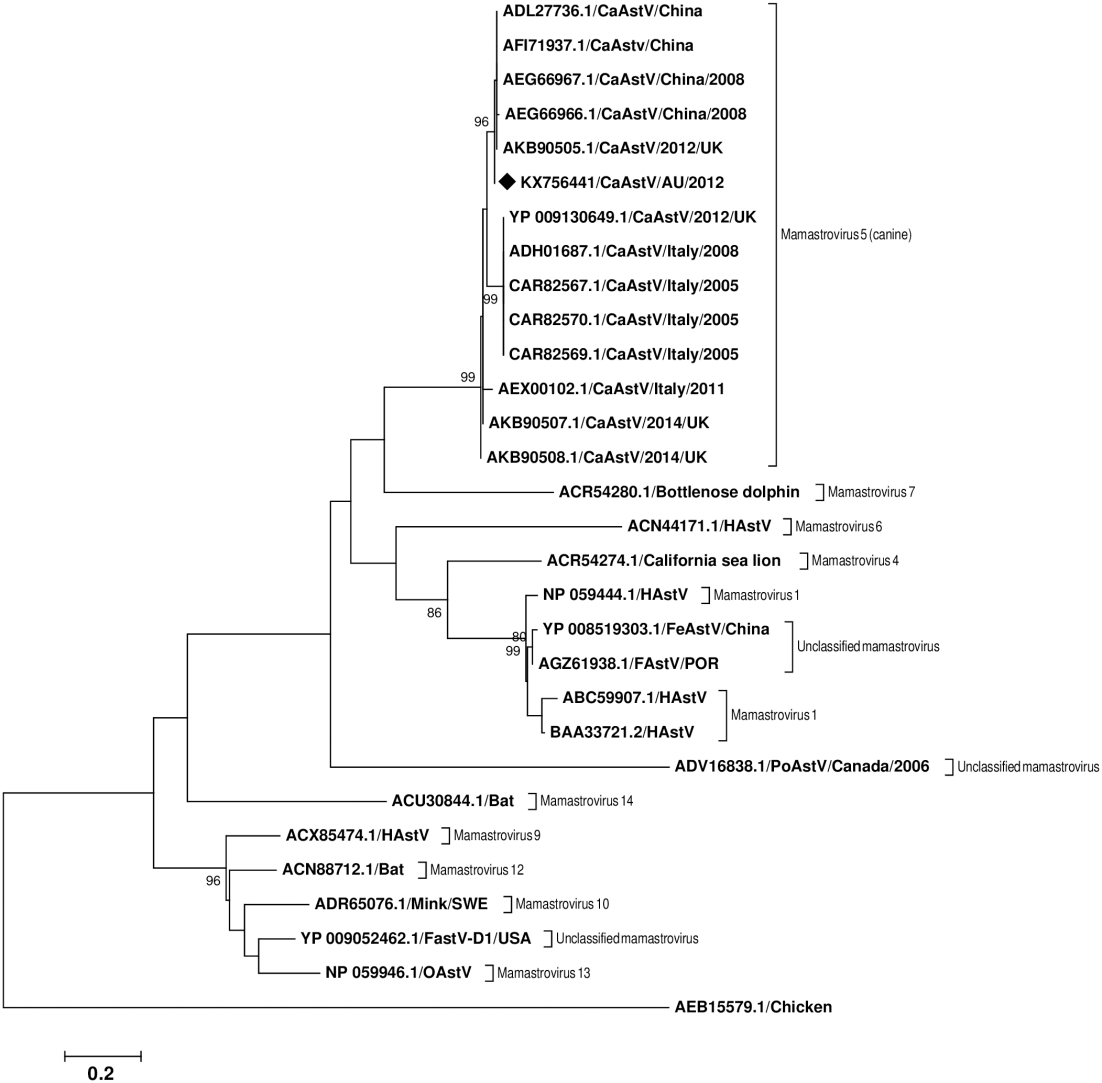
Phylogenetic analysis based on the conserved region of amino acid sequence of the capsid region of astroviruses from various mammalian species. GenBank accession numbers are shown for all sequences analysed and the sequence determined in this study is noted with a black diamond. The tree was constructed using the Maximum Likelihood method, based on the JTT matrix-based model with 1000 bootstrap replications. Bootstrap values ≥ 70% are indicated at each branch. Evolutionary analyses were conducted in MEGA6.

A phylogenetic tree was constructed using the protein alignment from the conserved region of the capsid (ORF2) of the astrovirus characterised in this study (DD1) and other canine astrovirus ORF2, together with *Mamastrovirus* sequences from different mammalian species, including a chicken astrovirus as an outgroup. The phylogenetic analysis grouped our canine astrovirus within the canine astrovirus clade. The closest canine astroviruses to our Australian sample were from UK and China with an identity between 98.82%–99.41% (Fig 2).

We also included the phylogenetic tree made with the full length of the ORF2 as a supplementary figure (S3 Fig).

## Discussion

Using next generation sequencing and metagenomics analysis, the virome in faecal samples from 8 healthy dogs and 8 dogs with acute diarrhoea is described. Only a single previous shotgun metagenomic study investigating the faecal virome of dogs with diarrhoea has been reported. In that study, mammalian viruses were found in 15 samples and two new virus species were described [6]. Our study analysed 16 faecal samples from dogs (8 healthy and 8 diarrhoeic), and identified eukaryotic viruses in 12 samples, including all diarrhoeic samples and 50% of the healthy samples. Thus, 70% of canine faeces contained eukaryotic viruses, suggesting that mammalian viruses are a common component of the enteric microbial population in dogs.

Our results must be interpreted with caution, due to bias created by SISPA. Areas of exaggerated depth appear when the SISPA method is used, creating artefacts during *de novo* assembly. This results in regions of repetitive sequences [36]. In order to overcome this bias, all contigs/singletons were analysed at family level and only for viral eukaryotic families were the results further analysed to evaluate what percentage they covered to some specific viral species.

The most common viral contigs/singletons identified in both groups were bacteriophages, similar to previous findings from human and other animal faecal virome studies [2, 5, 16, 19, 22, 27, 30]. Bacteriophages belonging to families *Myoviridae*, *Siphoviridae* and *Podoviridae* (dsDNA viruses from order *Caudovirales*) and ssDNA family *Microviridae* were identified, which is similar to other studies on faeces from humans [2, 5, 16], cats [31], horses [29], sea lions [24], pine martens and European badgers [26], ferrets [21] and small carnivores [11].

Bacteriophages modify diversity of bacterial populations due to their lytic life cycle and also promote different characteristics in the bacterial population due to their lysogenic life cycle transferring genes such as encoding toxins or resistance to antibiotic [38]. This life-cycle may lead to bacteriophages conferring advantage to some bacterial species in the environmental niche [39]. Therefore, it is possible that the greater amount of contigs/singletons corresponding to bacteriophages identified in the group of dogs with acute diarrhoea, when compared to healthy dogs, means a higher amount of bacteriophages. If so, bacteriophages could have generated a change in the normal balance of bacterial population resulting in dysbiosis, and ultimately causing diarrhoea. Conversely, it could be that an initial change in the bacterial population in these dogs resulted from the acute diarrhoea [40, 41] is the cause of the variation in the bacteriophage population. In our sample population, the latter explanation is most likely, because in a shelter environment a higher number of circulating pathogens, changes in diet and a stressful environment could contribute to the dysbiosis associated with acute diarrhoea [42]. The bacterial microbiome and the analysis of contigs/singletons matching specific bacteriophages were not assessed in this study, therefore further microbiome/virome cross analysis is necessary to elucidate the association between bacteria and bacteriophages in dogs. However, even this analysis would be unlikely to determine the cause or effect relationship between bacteriophages and dysbiosis at a single point in time.

The analysis of the lowest common ancestor of eukaryotic viral families, according to MEGAN, identified eight eukaryotic virus species (Table 1). However, each of these results require validation by targeted PCR, or whole genome characterisation of each species, as the NGS results after SISPA amplification may be biased and not accurate depiction at a species level [36].

Sequences matching those of human viruses (adenovirus and papillomavirus) were found in one sample from a healthy dog. Only one contig from each virus covering a very small percentage of the genome in both cases. This finding could suggest contamination during collection or processing.

Known enteric pathogenic families *Parvoviridae* and *Coronaviridae* were identified in samples from both healthy dogs and dogs with acute diarrhoea. Interestingly, almost all positive samples were from puppies (between 4–8 months) that had been vaccinated less than one month prior to sampling. The lowest common ancestry analysis in MEGAN of the contigs/singletons matching *Parvoviridae* family, suggested they were canine parvovirus (CPV), but as CPV positive dogs (as tested by faecal antigen tests) were not included in this study it is highly likely these results represent vaccine derived sequences not detected by the CPV antigen detection kit, or represent a virus load below the level of detection. Previous studies have demonstrated that modified live vaccine virus can be detected in faecal samples for extended periods of time after vaccination [43]. Further genome characterisation of these canine parvovirus is warranted to confirm this hypothesis.

Three individual samples contained *Coronaviridae* contigs/singletons, two of which were from puppies [ND10 and DD8] (Table 1) and one from an adult dog [DD1] (Table 1). Our results are consistent with Li et al 2011, who also reported the highest number of *Coronaviridae* reads in one sample collected from a puppy [6]. Canine coronavirus can be shed in faeces in high numbers for up to 156 days [44, 45]. These findings validate the affinity of the *Coronaviridae* viral family to infect young individuals [46], and present as a common enteric pathogen in a shelter environment [42, 45, 47].

The uncommon viruses, canine kobuvirus and canine norovirus, were identified only in samples from dogs with acute diarrhoea. Previous studies have suggested these viruses may have some association with enteric disease in dogs, however, both viral species have been detected in both healthy dogs and dogs with diarrhoea [6, 48]

Our shotgun metagenomic sequence data indicated that the most frequent RNA viral family in dog samples with acute diarrhoea was *Astroviridae*, being identified in more than half of the diarrheal samples. [49]. In dogs, astrovirus has been previously detected mainly in puppies with diarrhoea, but has also been occasionally reported in healthy dogs [50–54]. The only previous report of a possible canine astrovirus in Australia was described in canine faeces in the 1984, where astrovirus–like particles were detected using electronic microscopy in healthy dogs [55].

To date, canine astrovirus has been reported in USA [56], China [51], Italy [50, 57, 58], UK [52], France [53], Brazil [59], Korea [60] and Japan [54]. The first description of the complete genome of two canine astroviruses was reported by a group of researchers from the UK in 2015 [52]. The current study contributes the first description of the complete genome of a canine astrovirus identified in Australia. In our study, using Sanger sequencing a near complete genome of a canine astrovirus was assembled from one dog with acute diarrhoea. A phylogenetic tree, analysing the capsid region (ORF2) of this Australian canine astrovirus and other astrovirus sequences present in GenBank, determined that it belonged to the canine astrovirus clade, very closely related to the canine astrovirus strains from the UK and China (Fig 2).

It is interesting to note that all canine astrovirus positive samples, were collected from the same shelter and obtained within a short period of time (Sept–Nov 2012). We could infer that this virus was endemic at that time in that shelter, and or maybe could represent an outbreak of diarrhoea in the shelter within that period of time. A more sensitive test (i.e.: quantitative PCR) in a larger number of samples from cases and controls may be useful to better understand the potential role of astroviruses as an aetiological agent in acute diarrhoea of dogs.

## Conclusion

In this study we analysed the faecal virome in healthy dogs and compared these findings with the faecal virome of dogs with acute diarrhoea. Known DNA and RNA viruses were found, together with different proportions of bacteriophages in each group. In addition, we described and characterised the first complete genome of a canine astrovirus in Australia. Future longitudinal studies analysing viruses, bacteria and other potential pathogens should be performed to assess the aetiology of diarrhoea in dogs and further elucidate the pathological importance of viruses found in dog intestines.

## Material and methods

### Animals and sample collection

Faecal samples from a total of 16 dogs were obtained between September 2012 and March 2013. All dogs were aged between 2.5 months and 7 years; and comprised 5 females and 11 males of various breeds (Table 1).

All faecal samples were collected from a single shelter in Melbourne (Lost Dogs Home), Australia. All samples were maintained at 4°C for up to 24 hrs, then were transported on dry ice before storing up to five aliquots of 500 mg of faeces each at -80°C until further analyses. Information about age, sex, breed, diet, vaccination and deworming status was recorded for each dog (University of Melbourne Animal Ethics Committee approval IDs 1413272.2 and 1112035.1).

Animals were determined to be healthy based on physical examination by a veterinarian and absence of any clinical signs of disease. Faecal consistency was considered normal as per published criteria (Faecal scoring chart, PURINA), and all dogs had been treated with deworming drugs for prophylaxis (Ilium Pyraquantal, TROY or Milbemax, Novartis). All samples were lifted from the floor, first thing in the morning before cleaning, during November 2012.

Faecal samples from 8 dogs with an acute onset of diarrhoea (less than 3 days of duration), were collected by a veterinarian from within the animal’s enclosure. All dogs with acute diarrhoea were tested for the presence of canine parvovirus antigen in faeces using the Anigen rapid CPV/CCV Ag Test kit, (Bionote). Positive samples were excluded from the study.

None of the dogs had been treated with antimicrobial drugs within the previous 8 weeks of sample collection. The majority of healthy dogs were receiving commercial dry food and some of the dogs with diarrhoea were being fed a high-fibre prescription veterinary diet (Hill’s i/d diet).

### Sample preparation and faecal extract preparation

Faecal samples were processed as described previously [6, 12]. Briefly, aliquots of 500 mg of faecal sample were thawed and re-suspended in saline buffer (0.01M Tris solution (pH7.5), 0.15M NaCl, 0.01M CaCl_2_) at 3:1 ratio of solid mass. One mm zirconia/silica beads were added to the stool solution, filling around 150μL of an Eppendorf tube, and vortexed vigorously for 3 minutes. The samples were then centrifuged at 17900 x *g* for 5 min, collecting the supernatant and repeating this step three more times. To reduce solid faecal matter and bacterial contamination, 500 μl of this solution was filtered through a 0.45 μm tube filter (Corning Costar Spin X) by centrifugation at 3800 x *g* for 5 minutes, then the filtrate was transferred to 2 mL tubes.

### Pre-extraction nucleic acid digestion

To enrich for viral DNA and RNA, a DNase/RNase step was incorporated using a modified protocol described previously [6, 12]. Each sample was treated with a cocktail of DNases (Turbo DNase, from Ambion, Baseline-ZERO from Epicentre, Benzonase from Novagen and DNase I from Roche) and RNase A (QIAGEN). This mixture was incubated in a water bath at 37°C for 3 hours. To stop the enzymatic activity, EDTA (AMRESCO) was added in a final concentration of 15 mM to each sample and incubated at 75°C for 10 min.

### Nucleic acid extraction and reverse transcription

Viral DNA/RNA protected from digestion within viral capsids were extracted using QIAamp Viral RNA mini kit (QIAGEN), according to manufacturer’s recommendations. A second DNase/RNase step was performed on the extracted viral RNA for elimination of genomic DNA, using DNase I recombinant, RNase free (10U/μl) (Roche) and Protector RNase inhibitor (40 U/μl) (Roche). After digestion of the DNA, the viral RNA was transcribed with Sensiscript Reverse Transcriptase kit (QIAGEN; Sensiscript RT kit) to generate cDNA, according to manufacturer’s instructions with minor modifications. Briefly, for a more sensitive detection in the subsequent PCR, a mixture of oligo-dt primers (Oligo (dT)15 primer, Promega) and random primers (Random hexamers, TaqMan Reverse Transcription Reagents, Roche, Applied Biosystems) were used and a RNA denaturation step (95° for 3 minutes) was added.

### Random amplification, Sequence-Independent Single Primer Amplification (SISPA) method

Viral cDNA and genomic DNA were randomly amplified using a modified SISPA protocol [61, 62]. Briefly, a second strand synthesis was performed with Large (Klenow) Fragment (New England Biolabs) and random hexamers (Roche, Biosystems, 50μM) followed by digestion of the second strand product with the restriction enzyme *CviQI* (Csp6.1), (New England Biolabs). Then a *CSp11*/*NBam24* adaptor was ligated to the digested DNA using T4 DNA ligase (Invitrogen) followed by PCR amplification of the adaptor-ligated product with NBam24 PCR primers. An aliquot of the PCR product was validated on a 1% agarose TBE gel, where a positive smear with multiple bands confirmed the random SISPA amplification of nucleic acid products.

### Viral library preparation and sequencing

The amplified PCR products were cleaned up using WIZARD SV Gel and PCR clean-up system (Promega) following manufacturer’s recommendations and two libraries with dual indexing for each sample were generated (DNA and cDNA) with Illumina Nextera XT DNA Sample Preparation kit, according to manufacturer indications. After visualise it with Agilent 2200 Tape Station System (Agilent Technologies), the libraries were submitted to the Australian Genome Research Facility (AGRF) for a 250 bases paired-end sequencing on the MiSeq Illumina platform.

### Bioinformatic analyses

All raw sequences were deposited under Bioproject ID: PRJNA380672 at NCBI database. Raw sequences were trimmed by quality score with PrinSeq software (v0.20.3) [63], filtering for low quality reads from both ends using the DUST score [64] with a threshold of 7. Poly A/T tails in both ends (ten nucleotides of each end) and SISPA primers sequences were also removed using this software. The Mothur software v.1.31.2 [65] was applied and the sequences were trimmed again, eliminating homopolymers, ambiguous bases and sequences less than 100bp. After these trimming steps, high quality reads (HQR) were obtained and all bad quality reads were removed from the group file (Fig 1).

The HQR were then compared against a dog chromosome database (CanFam3.1) using the BLASTn (Blast 2.2.29+ standalone) algorithm with an 80% identity cut off. The BLASTn files were analysed by MEGAN V5.2.1 [37] and all dog sequences were removed using Mothur v.1.31.2.

Subsequently, these dog free sequences were compared against a bacterial database (CAMERA prokaryotic nucleotide database 10572.V7, Nov 2012; http://camera.calit2.net/) [66] to eliminate bacterial sequences, using the BLASTn (Blast 2.2.29+ standalone) algorithm with an 80% identity cut off. To extract cellular organism sequences from the group file, MEGAN V5.2.1 and Mothur software were used as described above (Fig 1).

The host and bacteria free sequence reads, were *de novo* assembled with MetaVelvet (velvet 1.2.08, KMER51) [67] using Kmer size 51 and contigs and singletons were created. These singletons were clustered with a 98% similarity using CD-HIT-est (version.4.5.4 2011) [68] (Fig 1).

All contigs and singletons clusters were analysed through two pipelines. (1) Contigs and singletons clusters were compared against the CAMERA Viral Nucleotide Sequence database 10570.V9, using tBLASTx search with an E-value cut off 10^−5^; (2) Contigs and singletons clusters were compared against the NCBI nucleotide database (2012) using BLASTn search with an E value cut off 1. All blast searches were performed in Blast 2.2.29+ standalone. These files were then analysed by MEGAN V5.2.1 [37] and the lowest common ancestor of known viral sequences were identified (Fig 1).

Finally, all viral contigs and singletons of eukaryotic organisms present in both analyses were aligned and compared with the NCBI reference sequence of the lowest common ancestor given by MEGAN V5.2.1. All alignments were made using Sequencher version 5.0.1 sequence analysis software (Gene Codes Corporation, Ann Arbor, MI USA) with minimum match percentage of 70%–80% and minimum overlap 50 as assembly parameters, evaluating the percentage of coverage of the genome.

All contigs and singletons with no hits were re-evaluated using online BLASTn with an E value cut off 10 and visualised with MEGAN V5.2.1 to evaluate the alignment with its lowest common ancestor.

### Sequencing of canine astrovirus genome

In order to acquire the complete genome of canine astrovirus, multiple sets of primers were selected from the literature or designed based on sequences obtained from Illumina reads (S2 Table). Nucleic acids from a single faecal sample from a dog with acute diarrhoea (DD1), which had 18 contigs/singletons of canine astrovirus (after tBLASTx analysis) was used to determine the complete genome sequence. RNA was extracted directly from the centrifuged sample after faecal extraction, previous to enrichment of viral nucleic acids as outlined before.

RT-PCR was performed with SuperScript III One-Step RT-PCR System with Platinum Taq (Invitrogen^™^). PCR conditions used were: 45°C for 60 min and 95°C for 5 min, 35 cycle of 94°C for 40 sec, 55°C for 1min and 72°C for 5 min, and a final elongation step of 72°C for 5 min, followed by final hold at 4°C. PCR products were run on a 1.2% agarose TBE gel stained with RedSafe^™^ nucleic acid staining solution (iNtRON Biotechnology). All PCR products were excised and cleaned up with WIZARD SV Gel and PCR clean-up system (Promega) following manufacturer’s protocol and sequenced using Sanger sequencing at the AGRF.

The near complete genome of the canine astrovirus was assembled using Sequencher version 5.0.1 sequence analysis software (Gene Codes Corporation, Ann Arbor, MI USA) with minimum match percentage 80 and minimum overlap 50 as assembly parameters.

### Phylogenetic analysis

Phylogenetic analysis of this canine astrovirus was performed aligning protein sequences of the 172 conserved amino acids of the capsid region (ORF2) from different species (S2 Fig), using CLUSTAL W, from MEGA version 6.0 [69] with default settings. A phylogenetic tree with 1000 bootstrap was generated using the Maximum likelihood method based on the JTT matrix-based model [70], using MEGA version 6.0. The percentage of identity was calculated with CLUSTALO 1.2.4 [71]

## Supporting information

S1 Fig MEGAN taxonomic tree showing distribution and number of contigs/singletons in each sample. (PDF)

S2 Fig Multiple alignment of ORF2, conserved region. Disagreements to consensus sequence are highlighted.
(PDF)

S3 Fig Phylogenetic analysis based on the full length amino acid sequence of the capsid region of astroviruses from various mammalian species. (PDF)

S1 Table Number of reads at each step during the bioinformatic pipeline; number and classification of contigs/singletons; and minimum (Min), mean and maximum (Max) size of contigs/singletons for each eukaryotic viral family. (PDF)

S2 Table Oligonucleotides used in characterisation of canine astrovirus. (PDF)

## References

[1] Reyes A, Haynes M, Hanson N, Angly F, Heath A, Rohwer F, et al Viruses in the faecal microbiota of monozygotic twins and their mothers. Nature. 2010;466(7304):334–8. 10.1038/nature09199 20631792 PMC2919852

[2] Breitbart M, Hewson I, Felts B, Mahaffy JM, Nulton J, Salamon P, et al Metagenomic analyses of an uncultured viral community from human feces. Journal of Bacteriology. 2003;185(20):6220–3. 10.1128/JB.185.20.6220-6223.2003 14526037 PMC225035

[3] Delwart EL. Viral metagenomics. Reviews in Medical Virology. 2007;17(2):115–31. 10.1002/rmv.532 17295196 PMC7169062

[4] Blomström A-L. Viral metagenomics as an emerging and powerful tool in veterinary medicine. Veterinary Quarterly. 2011;31(3):107–14. 10.1080/01652176.2011.604971 22029881

[5] Breitbart M, Haynes M, Kelley S, Angly F, Edwards RA, Felts B, et al Viral diversity and dynamics in an infant gut. Research in Microbiology. 2008;159(5):367–73. 10.1016/j.resmic.2008.04.006 18541415

[6] Li LL, Pesavento PA, Shan TL, Leutenegger CM, Wang CL, Delwart E. Viruses in diarrhoeic dogs include novel kobuviruses and sapoviruses. J Gen Virol. 2011;92:2534–41. 10.1099/vir.0.034611-0 . 21775584 PMC3352364

[7] Li L, Victoria J, Wang C, Jones M, Fellers G, Kunz T, et al Bat guano virome: predominance of dietary viruses from insects and plants plus novel mammalian viruses. Journal of virology. 2010;84(14):6955–65. 10.1128/JVI.00501-10 20463061 PMC2898246

[8] Minot S. The human gut virome: Inter-individual variation and dynamic response to diet. Genome research. 2011;21(10):1616–25. 10.1101/gr.122705.111 21880779 PMC3202279

[9] Shan T, Li L, Simmonds P, Wang C, Moeser A, Delwart E. The fecal virome of pigs on a high-density farm. Journal of virology. 2011;85(22):11697–708. 10.1128/JVI.05217-11 21900163 PMC3209269

[10] Ng TFF, Mesquita JR, Nascimento MSJ, Kondov NO, Wong W, Reuter G, et al Feline fecal virome reveals novel and prevalent enteric viruses. Vet Microbiol. 2014;171(1–2):102–11. 10.1016/j.vetmic.2014.04.005. 24793097 PMC4080910

[11] Bodewes R, Ruiz Gonzalez A, Schapendonk C, van den Brand JMA, Osterhaus A, Smits S. Viral metagenomic analysis of feces of wild small carnivores. Virology journal. 2014;11(1):89. 24886057 10.1186/1743-422X-11-89 PMC4030737

[12] Victoria JG, Kapoor A, Li LL, Blinkova O, Slikas B, Wang CL, et al Metagenomic analyses of viruses in stool samples from children with acute flaccid paralysis. Journal of Virology. 2009;83(9):4642–51. 10.1128/JVI.02301-08 . 19211756 PMC2668503

[13] Zhang T, Breitbart M, Lee WH, Run J-Q, Wei CL, Soh SWL, et al RNA viral community in human feces: prevalence of plant pathogenic viruses. PLoS Biol. 2005;4(1):e3. 10.1371/journal.pbio.0040003 PMC1310650 16336043

[14] Finkbeiner SR, Allred AF, Tarr PI, Klein EJ, Kirkwood CD, Wang D. Metagenomic analysis of human diarrhea: viral detection and discovery. PLoS Pathogens. 2008;4(2):1–9. 10.1371/journal.ppat.1000011 PMC2290972 18398449

[15] Pérez-Brocal V, García-López R, Vázquez-Castellanos JF, Nos P, Beltrán B, Latorre A, et al Study of the viral and microbial communities associated with Crohn’s disease: a metagenomic approach. Clinical and translational gastroenterology. 2013;4(6):e36. 23760301 10.1038/ctg.2013.9 PMC3696940

[16] Norman J, Handley S, Baldridge M, Droit L, Liu C, Keller B, et al Disease-specific alterations in the enteric virome in inflammatory bowel disease. Cell. 2015;160(3):447–60. 10.1016/j.cell.2015.01.002 25619688 PMC4312520

[17] Holtz L, Cao S, Zhao G, Bauer I, Denno D, Klein E, et al Geographic variation in the eukaryotic virome of human diarrhea. Virology. 2014;468:556–64. 10.1016/j.virol.2014.09.012 25262473 PMC4254309

[18] Lim ES, Zhou Y, Zhao G, Bauer IK, Droit L, Ndao IM, et al Early life dynamics of the human gut virome and bacterial microbiome in infants. Nature Medicine. 2015;21(10):1228–34. 10.1038/nm.3950 http://www.nature.com/nm/journal/v21/n10/abs/nm.3950.html#supplementary-information. 26366711 PMC4710368

[19] Wagner J, Maksimovic J, Farries G, Sim W, Bishop R, Cameron D, et al Bacteriophages in gut samples from pediatric Crohn's disease patients: metagenomic analysis using 454 pyrosequencing. Inflammatory Bowel Diseases. 2013;19(8):1598–608. 23749273 10.1097/MIB.0b013e318292477c

[20] Minot S, Bryson A, Chehoud C, Wu G, Lewis J, Bushman F. Rapid evolution of the human gut virome. Proceedings of the National Academy of Sciences of the United States of America. 2013;110(30):12450–5. 10.1073/pnas.1300833110 23836644 PMC3725073

[21] Smits S, Raj VS, Oduber M, Schapendonk CME, Bodewes R, Provacia L, et al Metagenomic analysis of the ferret fecal viral flora. PLoS ONE. 2013;8(8):e71595 10.1371/journal.pone.0071595 23977082 PMC3748082

[22] Phan TG, Kapusinszky B, Wang C, Rose RK, Lipton HL, Delwart EL. The fecal viral flora of wild rodents. PLoS Pathog. 2011;7(9):e1002218 10.1371/journal.ppat.1002218 21909269 PMC3164639

[23] Phan TG, Vo NP, Boros Á, Pankovics P, Reuter G, Li OT, et al The viruses of wild pigeon droppings. PloS one. 2013;8(9):e72787 10.1371/journal.pone.0072787 24023772 PMC3762862

[24] Li L, Shan T, Wang C, Côté C, Kolman J, Onions D, et al The Fecal Viral Flora of California Sea Lions. Journal of Virology. 2011;85(19):9909–17. 10.1128/JVI.05026-11 21795334 PMC3196430

[25] Ge X, Li Y, Yang X, Zhang H, Zhou P, Zhang Y, et al Metagenomic analysis of viruses from bat fecal samples reveals many novel viruses in insectivorous bats in China. Journal of virology. 2012;86(8):4620–30. 10.1128/JVI.06671-11 22345464 PMC3318625

[26] van den Brand JMA, van Leeuwen M, Schapendonk CM, Simon JH, Haagmans BL, Osterhaus ADME, et al Metagenomic analysis of the viral flora of pine marten and european badger feces. Journal of Virology. 2012;86(4):2360–5. 10.1128/JVI.06373-11 22171250 PMC3302375

[27] Woo PCY, Lau SKP, Teng JLL, Tsang AKL, Joseph M, Wong EYM, et al Metagenomic analysis of viromes of dromedary camel fecal samples reveals large number and high diversity of circoviruses and picobirnaviruses. Virology. 2014;471–473(0):117–25. 10.1016/j.virol.2014.09.020. PMC7112128 25461537

[28] Haynes M, Rohwer F. The Human Virome In: Nelson KE, editor. Metagenomics of the Human Body: Springer New York; 2011 p. 63–77.

[29] Cann A, Fandrich S, Heaphy S. Analysis of the virus population present in equine faeces indicates the presence of hundreds of uncharacterized virus genomes. Virus genes. 2005;30(2):151–6. 10.1007/s11262-004-5624-3 15744573

[30] Zhang B, Tang C, Yue H, Ren Y, Song Z. Viral metagenomics analysis demonstrates the diversity of viral flora in piglet diarrhoeic faeces in China. J Gen Virol. 2014;95(7):1603–11. 24718833 10.1099/vir.0.063743-0

[31] Zhang W, Li L, Deng X, Kapusinszky B, Pesavento PA, Delwart E. Faecal virome of cats in an animal shelter. J Gen Virol. 2014;95(Pt 11):2553–64. 10.1099/vir.0.069674-0 25078300 PMC4202271

[32] Lappin M. Chapter 57—Small Intestine In: Washabau RJ, Day MJ, editors. Canine and Feline Gastroenterology. Saint Louis: W.B. Saunders; 2013 p. 651–728.

[33] Schulz BS, Strauch C, Mueller RS, Eichhorn W, Hartmann K. Comparison of the prevalence of enteric viruses in healthy dogs and those with acute haemorrhagic diarrhoea by electron microscopy. Journal of Small Animal Practice. 2008;49(2):84–8. 10.1111/j.1748-5827.2007.00470.x . 18086157 PMC7166461

[34] Tupler T, Levy JK, Sabshin SJ, Tucker SJ, Greiner EC, Leutenegger CM. Enteropathogens identified in dogs entering a Florida animal shelter with normal feces or diarrhea. Journal Of The American Veterinary Medical Association. 2012;241(3):338–43. 10.2460/javma.241.3.338 . 22812470

[35] Yesilbag K, Yilmaz Z, Ozkul A, Pratelli A. Aetiological role of viruses in puppies with diarrhoea. Veterinary Record. 2007;161(5):169–70. . 17675636 10.1136/vr.161.5.169

[36] Rosseel T, Van Borm S, Vandenbussche F, Hoffmann B, van den Berg T, Beer M, et al The origin of biased sequence depth in sequence-independent nucleic acid amplification and optimization for efficient massive parallel sequencing. PloS one. 2013;8(9):e76144 10.1371/journal.pone.0076144 24086702 PMC3784409

[37] Huson DH, Mitra S, Ruscheweyh H-J, Weber N, Schuster SC. Integrative analysis of environmental sequences using MEGAN4. Genome Research. 2011;21(9):1552–60. 10.1101/gr.120618.111 21690186 PMC3166839

[38] De Paepe M, Leclerc M, Tinsley CR, Petit M-A. Bacteriophages: an underestimated role in human and animal health? Frontiers in cellular and infection microbiology. 2014;4:39 10.3389/fcimb.2014.00039 24734220 PMC3975094

[39] Virgin HW. The virome in mammalian physiology and disease. Cell. 2014;157(1):142–50. 10.1016/j.cell.2014.02.032 24679532 PMC3977141

[40] Suchodolski JS. The fecal microbiome in dogs with acute diarrhea and idiopathic inflammatory bowel disease. PLoS ONE. 2012;7(12):e51907 10.1371/journal.pone.0051907 23300577 PMC3530590

[41] Guard B, Barr J, Reddivari L, Klemashevich C, Jayaraman A, Steiner J, et al Characterization of microbial dysbiosis and metabolomic changes in dogs with acute diarrhea. PLoS One. 2015;10(5):e0127259 10.1371/journal.pone.0127259 26000959 PMC4441376

[42] Pesavento PA, Murphy BG. Common and Emerging Infectious Diseases in the Animal Shelter. Veterinary pathology. 2013. 10.1177/0300985813511129 24265288

[43] Decaro N, Crescenzo G, Desario C, Cavalli A, Losurdo M, Colaianni ML, et al Long-term viremia and fecal shedding in pups after modified-live canine parvovirus vaccination. Vaccine. 2014;32(30):3850–3. 10.1016/j.vaccine.2014.04.050. 24793948 PMC7115601

[44] Pratelli A, Elia G, Martella V, Tinelli A, Decaro N, Marsilio F, et al M gene evolution of canine coronavirus in naturally infected dogs. The Veterinary Record. 2001;151(25):758–61. 12521247

[45] Decaro N, Buonavoglia C. An update on canine coronaviruses: Viral evolution and pathobiology. Vet Microbiol. 2008;132(3–4):221–34. 10.1016/j.vetmic.2008.06.007. 18635322 PMC7117484

[46] Evermann JF, Abbott JR, Han S. Canine coronavirus-associated puppy mortality without evidence of concurrent canine parvovirus infection. Journal of veterinary diagnostic investigation. 2005;17(6):610–4. 10.1177/104063870501700618 16475526

[47] Greene CE, Decaro N. Chapter 8 Canine Viral Enteritis In: Greene CE, editor. Infectious diseases of the dog and cat. 4th ed: Elsevier Health Sciences; 2013 p. 67–80.

[48] Mesquita JR, Barclay L, Nascimento MS, Vinje J. Novel norovirus in dogs with diarrhea. Emerg Infect Dis. 2010;16(6):980–2. 10.3201/eid1606.091861 20507751 PMC3086253

[49] Mendez E, Arias C. Astroviruses In: Knipe DM, Howley PM, editors. Fields virology,. 1. 6th ed Philadelphia: Wolters Kluwer/Lippincott Williams & Wilkins Health; 2013 p. 609–28.

[50] Martella V, Moschidou P, Lorusso E, Mari V, Camero M, Bellacicco A, et al Detection and characterization of canine astroviruses. J Gen Virol. 2011;92:1880–7. 10.1099/vir.0.029025-0 . 21471316

[51] Zhu AL, Zhao W, Yin H, Shan TL, Zhu CX, Yang X, et al Isolation and characterization of canine astrovirus in China. Archives of Virology. 2011;156(9):1671–5. 10.1007/s00705-011-1022-z 21604183

[52] Caddy S, Goodfellow I. Complete genome sequence of canine astrovirus with molecular and epidemiological characterisation of UK strains. Vet Microbiol. 2015;177(1–2):206–13. 10.1016/j.vetmic.2015.03.011 25818578 PMC4401448

[53] Grellet A, De Battisti C, Feugier A, Pantile M, Marciano S, Grandjean D, et al Prevalence and risk factors of astrovirus infection in puppies from French breeding kennels. Vet Microbiol. 2012;157(1–2):214–9. 10.1016/j.vetmic.2011.11.012 . 22304762

[54] Takano T, Takashina M, Doki T, Hohdatsu T. Detection of canine astrovirus in dogs with diarrhea in Japan. Archives of Virology. 2015;160(6):1549–53. 10.1007/s00705-015-2405-3 25824600 PMC7087093

[55] Marshall JA, Healey DS, Studdert MJ, Scott PC, Kennett ML, Ward BK, et al Viruses and virus-like particles in the faeces of dogs with and without diarrhoea. Australian veterinary journal. 1984;61(2):33–8. 6329156 10.1111/j.1751-0813.1984.tb07186.x PMC7159758

[56] Williams FP. Astrovirus-like, coronavirus-like, and parvovirus-like particles detected in the diarrheal stools of beagle pups. Archives of Virology. 1980;66(3):215–26. 6778459 10.1007/BF01314735 PMC7086831

[57] Toffan A, Jonassen CM, De Battisti C, Schiavon E, Kofstad T, Capua I, et al Genetic characterization of a new astrovirus detected in dogs suffering from diarrhoea. Vet Microbiol. 2009;139(1–2):147–52. 10.1016/j.vetmic.2009.04.031. 19477085 PMC7126621

[58] Martella V, Moschidou P, Catella C, Larocca V, Pinto P, Losurdo M, et al Enteric disease in dogs naturally infected by a novel canine astrovirus. Journal of Clinical Microbiology. 2012;50(3):1066–9. 10.1128/JCM.05018-11 . 22189118 PMC3295184

[59] Castro TX, Cubel Garcia RCN, Costa EM, Leal RM, Xavier MdPT, Leite JPG. Molecular characterisation of calicivirus and astrovirus in puppies with enteritis. Veterinary record. 2013;172(21):557. 10.1136/vr.101566 23605075

[60] Choi S, Lim S-I, Kim Y, Cho Y-Y, Song J-Y, An D-J. Phylogenetic analysis of astrovirus and kobuvirus in korean dogs. Journal of Veterinary Medical Science. 2014;76(8):1141–5. 10.1292/jvms.13-0585 24784439 PMC4155196

[61] Allander T, Emerson SU, Engle RE, Purcell RH, Bukh J. A virus discovery method incorporating DNase treatment and its application to the identification of two bovine parvovirus species. Proceedings of the National Academy of Sciences of the United States of America. 2001;98(20):11609–14. 10.1073/pnas.211424698 11562506 PMC58777

[62] Reyes GR, Kim JP. Sequence-independent, single-primer amplification (SISPA) of complex DNA populations. Molecular And Cellular Probes. 1991;5(6):473–81. . 1664049 10.1016/s0890-8508(05)80020-9

[63] Schmieder R, Edwards R. Quality control and preprocessing of metagenomic datasets. Bioinformatics. 2011;27(6):863–4. 10.1093/bioinformatics/btr026 21278185 PMC3051327

[64] Morgulis A, Gertz EM, Schäffer AA, Agarwala R. A Fast and Symmetric DUST Implementation to Mask Low-Complexity DNA Sequences. Journal of Computational Biology. 2006;13(5):1028–40. 10.1089/cmb.2006.13.1028 16796549

[65] Schloss P, Westcott S, Ryabin T, Hall J, Hartmann M, Hollister E, et al Introducing mothur: open-source, platform-independent, community-supported software for describing and comparing microbial communities. Applied and environmental microbiology. 2009;75(23):7537–41. 10.1128/AEM.01541-09 19801464 PMC2786419

[66] Sun S, Chen J, Li W, Altintas I, Lin A, Peltier S, et al Community cyberinfrastructure for Advanced Microbial Ecology Research and Analysis: the CAMERA resource. Nucleic Acids Research. 2011;39(Database issue):D546–51. 10.1093/nar/gkq1102 21045053 PMC3013694

[67] Namiki T, Hachiya T, Tanaka H, Sakakibara Y. MetaVelvet: an extension of Velvet assembler to de novo metagenome assembly from short sequence reads. Nucleic Acids Research. 2012;40(20):e155 10.1093/nar/gks678 22821567 PMC3488206

[68] Li W, Godzik A. Cd-hit: a fast program for clustering and comparing large sets of protein or nucleotide sequences. Bioinformatics. 2006;22(13):1658–9. 10.1093/bioinformatics/btl158 16731699

[69] Tamura K, Stecher G, Peterson D, Filipski A, Kumar S. MEGA6: Molecular Evolutionary Genetics Analysis Version 6.0. Molecular Biology and Evolution. 2013;30(12):2725–9. 10.1093/molbev/mst197 . 24132122 PMC3840312

[70] Jones DT, Taylor WR, Thornton JM. The rapid generation of mutation data matrices from protein sequences. Computer applications in the biosciences: CABIOS. 1992;8(3):275–82. 1633570 10.1093/bioinformatics/8.3.275

[71] Sievers F, Wilm A, Dineen D, Gibson TJ, Karplus K, Li W, et al Fast, scalable generation of high-quality protein multiple sequence alignments using Clustal Omega. Molecular Systems Biology. 2011;7(1). 10.1038/msb.2011.75 21988835 PMC3261699

