# Supplementary material for: Characterisation of the canine faecal virome in healthy dogs and dogs with acute diarrhoea using shotgun metagenomics
Source: PLoS One. 2017 Jun 1;12(6):e0178433. doi: 10.1371/journal.pone.0178433 (PMC5453527; doi:10.1371/journal.pone.0178433)
Supplement: S1 Fig — (PDF) [file pone.0178433.s001.pdf]

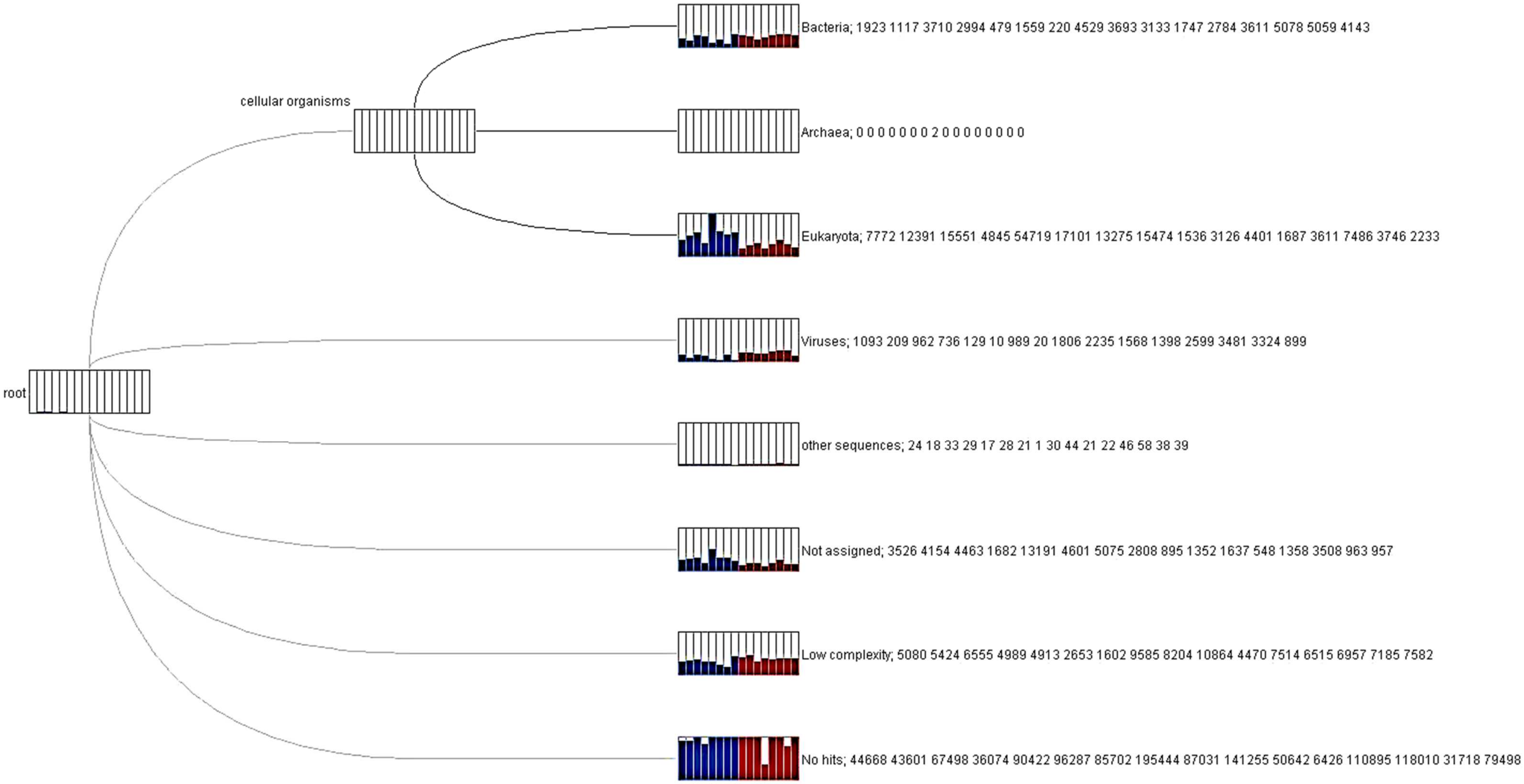

Figure S1: MEGAN taxonomic tree showing distribution and number of contigs/singletons in each sample. Numbers next to the charts indicate the numbers of contigs/singletons in each sample at every taxa. Root: contigs/singletons of all samples after *de novo* assembly. Blue columns: healthy samples, Red columns: dogs with acute diarrhoea samples
