# Supplementary material for: Characterisation of the canine faecal virome in healthy dogs and dogs with acute diarrhoea using shotgun metagenomics
Source: PLoS One. 2017 Jun 1;12(6):e0178433. doi: 10.1371/journal.pone.0178433 (PMC5453527; doi:10.1371/journal.pone.0178433)
Supplement: S3 Fig — (PDF) [file pone.0178433.s003.pdf]

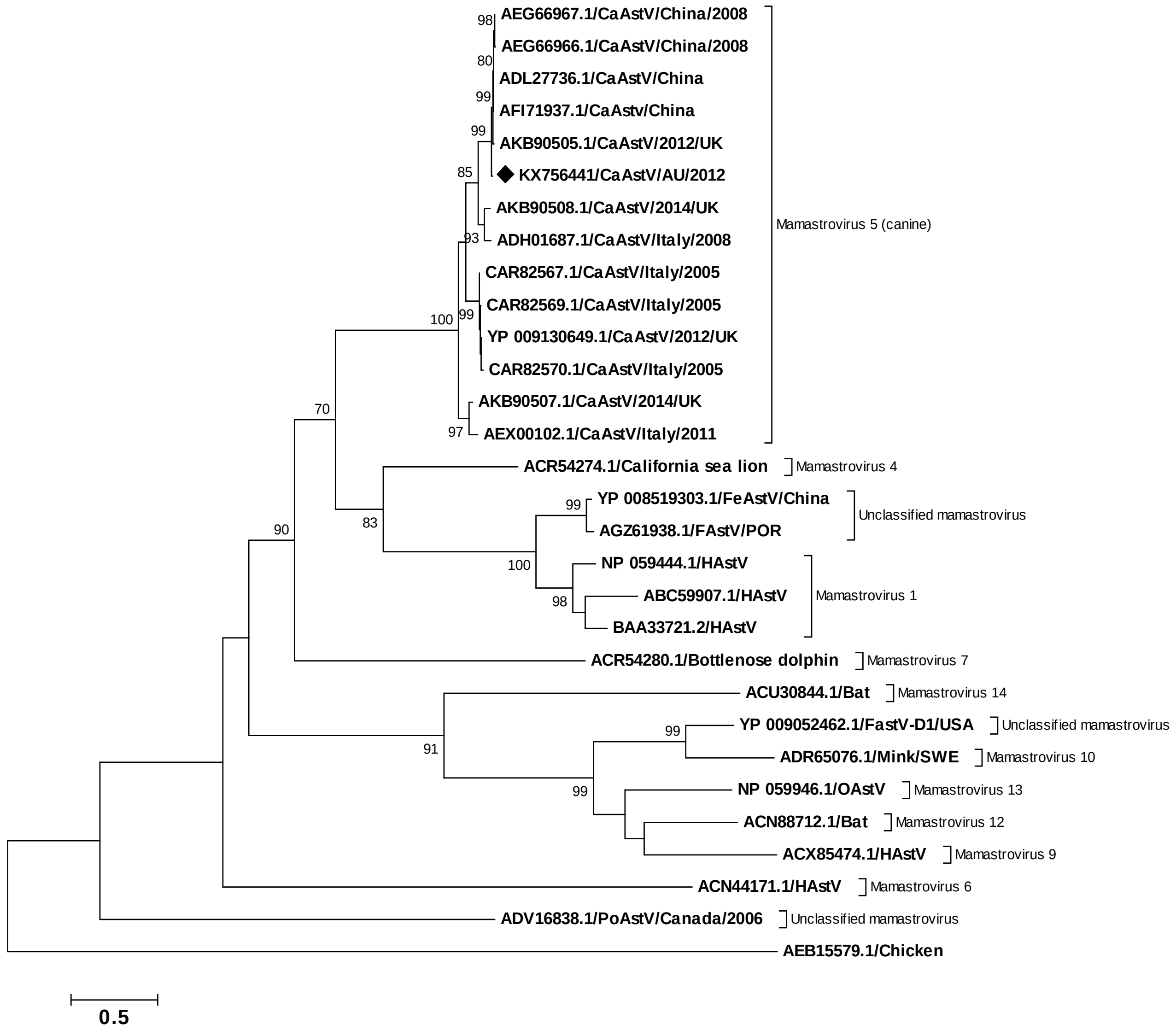

Figure S3: Phylogenetic analysis based on the full length amino acid sequence of the capsid region of astroviruses from various mammalian species. GenBank accession numbers are shown for all sequences analysed and the sequence determined in this study is noted with a black diamond. The tree was constructed using the Maximum Likelihood method, based on the JTT matrix-based model with 1000 bootstrap replications. Bootstrap values ≥70% are indicated at each branch. Evolutionary analyses were conducted in MEGA6.
