# Supplementary material for: Characterisation of the canine faecal virome in healthy dogs and dogs with acute diarrhoea using shotgun metagenomics
Source: PLoS One. 2017 Jun 1;12(6):e0178433. doi: 10.1371/journal.pone.0178433 (PMC5453527; doi:10.1371/journal.pone.0178433)
Supplement: S1 Table — (PDF) [file pone.0178433.s004.pdf]

Table S1: number of reads at each step during the bioinformatic pipeline; number and classification of contigs/singletons; and minimum (Min), mean and maximum (Max) size of contigs for each eukaryotic viral family

Sample name	Paired-end file names	Nº of raw sequences	High Quality Reads (HQR) Trimmed by quality	Dog sequences extracted	HQR cellular and viral sequences (Dog free sequences)	Cellular organisms sequences extracted	HQR viral sequences Dog and cell. org. free
**ND4**	ND4_DNA_R1	1311100	1134390	1605	1132785	923407	209378
	ND4_RNA_R1	1866166	1573342	3903	1569439	1353026	216413
	ND4_DNA_R2	1311100	1158643	1583	1157060	938453	218607
	ND4_RNA_R2	1866166	1547022	4489	1542533	1314009	228524
**ND5**	ND5_DNA_R1	1736359	1536868	3729	1533139	1249437	283702
	ND5_RNA_R1	1990867	1795532	4557	1790975	1611306	179669
	ND5_DNA_R2	1736359	1500716	3332	1497384	1206251	291133
	ND5_RNA_R2	1990867	1667959	4393	1663566	1480099	183467
**ND6**	ND6_DNA_R1	815204	743183	1434	741749	461093	280656
	ND6_RNA_R1	1645140	1493204	8841	1484363	1353584	130779
	ND6_DNA_R2	815204	750404	1701	748703	459550	289153
	ND6_RNA_R2	1645140	1347374	8192	1339182	1207044	132138
**ND7**	ND7_DNA_R1	1198122	1054259	117	1054142	934316	119826
	ND7_RNA_R1	1025856	952260	1207	951053	871329	79724
	ND7_DNA_R2	1198122	1082375	129	1082246	956680	125566
	ND7_RNA_R2	1025856	908242	1253	906989	826788	80201
**ND8**	ND8_DNA_R1	1465187	1314951	803	1314148	177130	1137018
	ND8_RNA_R1	1786048	1512166	147300	1364866	277672	1087194
	ND8_DNA_R2	1465187	1343643	808	1342835	148393	1194442
	ND8_RNA_R2	1786048	1568078	148497	1419581	245946	1173635
**ND9**	ND9_DNA_R1	1208162	1062399	1908	1060491	55277	1005214
	ND9_RNA_R1	1727061	1470574	85947	1384627	502996	881631
	ND9_DNA_R2	1208162	1065512	2031	1063481	25847	1037634
	ND9_RNA_R2	1727061	1428391	80566	1347825	425831	921994
**ND10**	ND10_DNA_R1	1164583	1004863	174	1004689	58792	945897
	ND10_RNA_R1	2065597	1820777	89560	1731217	788411	942806
	ND10_DNA_R2	1164583	1070826	169	1070657	33792	1036865
	ND10_RNA_R2	2065597	1706077	84174	1621903	643891	978012
**ND11**	ND11_DNA_R1	1657053	1392154	8164	1383990	514156	869834
	ND11_RNA_R1	1594165	1365243	18018	1347225	701964	645261
	ND11_DNA_R2	1657053	1383952	8381	1375571	441348	934223
	ND11_RNA_R2	1594165	1265384	18037	1247347	595568	651779
**DD1**	DD1_DNA_R1	927568	811717	129	811588	457413	354175
	DD1_RNA_R1	1403863	1249563	119	1249444	1231056	18388
	DD1_DNA_R2	927568	802403	132	802271	428809	373462
	DD1_RNA_R2	1403863	1201423	99	1201324	1190116	11208
**DD2**	DD2_DNA_R1	1377334	1207914	61	1207853	50603	1157250
	DD2_RNA_R1	1780885	1516340	274	1516066	1489247	26819
	DD2_DNA_R2	1377334	1145968	56	1145912	29040	1116872
	DD2_RNA_R2	1780885	1480746	313	1480433	1461423	19010
**DD3**	DD3_DNA_R1	490858	426251	160	426091	193750	232341
	DD3_RNA_R1	955369	857384	603	856781	831042	25739
	DD3_DNA_R2	490858	422577	174	422403	184050	238353
	DD3_RNA_R2	955369	848432	696	847736	826371	21365
**DD5**	DD5_DNA_R1	1052108	951196	79	951117	927185	23932
	DD5_RNA_R1	1544169	1323961	369	1323592	1299445	24147
	DD5_DNA_R2	1052108	860548	69	860479	842992	17487
	DD5_RNA_R2	1544169	1328612	416	1328196	1309927	18269
**DD7**	DD7_DNA_R1	1565728	1308876	724	1308152	673563	634589
	DD7_RNA_R1	2101608	1803874	287	1803587	1773208	30379
	DD7_DNA_R2	1565728	1189953	724	1189229	581705	607524
	DD7_RNA_R2	2101608	1679440	292	1679148	1655439	23709
**DD8**	DD8_DNA_R1	1629252	1366799	1688	1365111	682270	682841
	DD8_RNA_R1	2127325	1885864	472	1885392	1846841	38551
	DD8_DNA_R2	1629252	1294015	1545	1292470	568200	724270
	DD8_RNA_R2	2127325	1769648	426	1769222	1740760	28462
**DD9**	DD9_DNA_R1	1385737	1251037	114	1250923	1151356	99567
	DD9_RNA_R1	1961904	1755877	662	1755215	1713698	41517
	DD9_DNA_R2	1385737	995565	97	995468	900714	94754
	DD9_RNA_R2	1961904	1576436	639	1575797	1541926	33871
**DD10**	DD10_DNA_R1	900679	815499	439	815060	416921	398139
	DD10_RNA_R1	1411255	1281734	338	1281396	1256344	25052
	DD10_DNA_R2	900679	767363	413	766950	362251	404699
	DD10_RNA_R2	1411255	1216535	347	1216188	1200225	15963
**Total**		93744624	80414313	757958	79656355	53601276	26055079

Sample name	Nº total contigs/singletons *de novo* assembly R1* + R2[§] + DNA + RNA	Nº total contigs/singletons having no hits	Nº total viral contigs/ singletons	Nº prokaryotic viral contigs/ singletons	Nº eukaryotic viral contigs/ singletons	Contig sizes of eukaryotic viral families: Adenoviridae			Papillomaviridae			Reoviridae			Parvoviridae			Astroviridae			Caliciviridae			Coronaviridae			Picornaviridae		
						Min	mean	Max	Min	mean	Max	Min	mean	Max	Min	mean	Max	Min	mean	Max	Min	mean	Max	Min	mean	Max	Min	mean	Max
**ND4**	64004	44668	1051	1012	39							101	251.00	865															
**ND5**	66919	43601	209	209	0																								
**ND6**	98776	67498	953	953	0																								
**ND7**	51350	36074	731	731	0																								
**ND8**	163899	90422	82	82	0																								
**ND9**	122239	96287	8	5	3										251	251	251												
**ND10**	106884	85702	914	2	912																			101	209	251			
**ND11**	227873	195444	20	18	2	129	129	129	192	192	192																		
**DD1**	103195	87031	1798	1777	21													112	199	263							154	184	221
**DD2**	162010	141255	2223	2179	44										101	240	519	193	270	365									
**DD3**	64487	50642	1568	1559	9							208	228	248				193	220	244				113	236	292			
**DD5**	20379	6426	1398	1387	11													199	219	251	196	267	493						
**DD7**	128635	110895	2596	2593	3							125	191	251															
**DD8**	144578	118010	3444	3230	214										242	248	251							101	222	380			
**DD9**	52034	31718	3324	3319	5							138	216	251															
**DD10**	95353	79498	891	886	5							202	284	433				126	126	126									
**Total**	1672615	1285171	21210	19942	1268																								

* R1: paired end 1
§ R2: paired end 2
