# Supplementary material for: Characterisation of the canine faecal virome in healthy dogs and dogs with acute diarrhoea using shotgun metagenomics
Source: PLoS One. 2017 Jun 1;12(6):e0178433. doi: 10.1371/journal.pone.0178433 (PMC5453527; doi:10.1371/journal.pone.0178433)
Supplement: S2 Table — (PDF) [file pone.0178433.s005.pdf]

Table S2: Oligonucleotides used in characterisation of canine astrovirus

Oligonucleotide	Sequence	Reference
625F-1	GTACTATACCRTTCTGATTTAATT	Martella et al 2011
626R-1	AGACCAARGTGTCATAGTTCAG	Martella et al 2011
501F20	CTAACAATCGTGGTCGCAAG	Grellet 2012
1156R21	TTGATTTGTGCATCCTTGTCC	Grellet 2012
ORF1a	TGAAGGACTGCTCAGAGTG	Caddy 2015
PM_ASTRO_DD1_1F	CCAAGAGTTGGTTTGGGTGATTAAA	This study
PM_ASTRO_DD1_349R	GCACTCTGAGCAGTCCTTCA	This study
PM_ASTRO_DD1_1201F	CTGATGTCCTCTGTCGGTAACAA	This study
PM_ASTRO_DD1_3185R	TGCTCCGGACATAATCCTTGAA	This study
PM_ASTRO_DD1_3527F	TGCCGAACAGAGGAGGAAAT	This study
PM_ASTRO_DD1_4908R	GGGAGCATTCTGGTAGGTGG	This study
PM_ASTRO_DD1_4357F	CGTGGTCGCAAGAGAGTTGA	This study
PM_ASTRO_DD1_5854R	AGTTGGAGTAGCGTATCTGGC	This study
PM_ASTRO_DD1_5525F	AATACCAGCAGAACCCACCG	This study
PM_ASTRO_DD1_6526R	AAAAGAAAAGAGTGAAAGTGAACCT	This study
