# Supplementary material for: Characterisation of the canine faecal virome in healthy dogs and dogs with acute diarrhoea using shotgun metagenomics
Source: PLoS One. 2017 Jun 1;12(6):e0178433. doi: 10.1371/journal.pone.0178433 (PMC5453527; doi:10.1371/journal.pone.0178433)
Supplement: S2 Fig — Disagreements to consensus sequence are highlighted. (PDF) [file pone.0178433.s002.pdf]

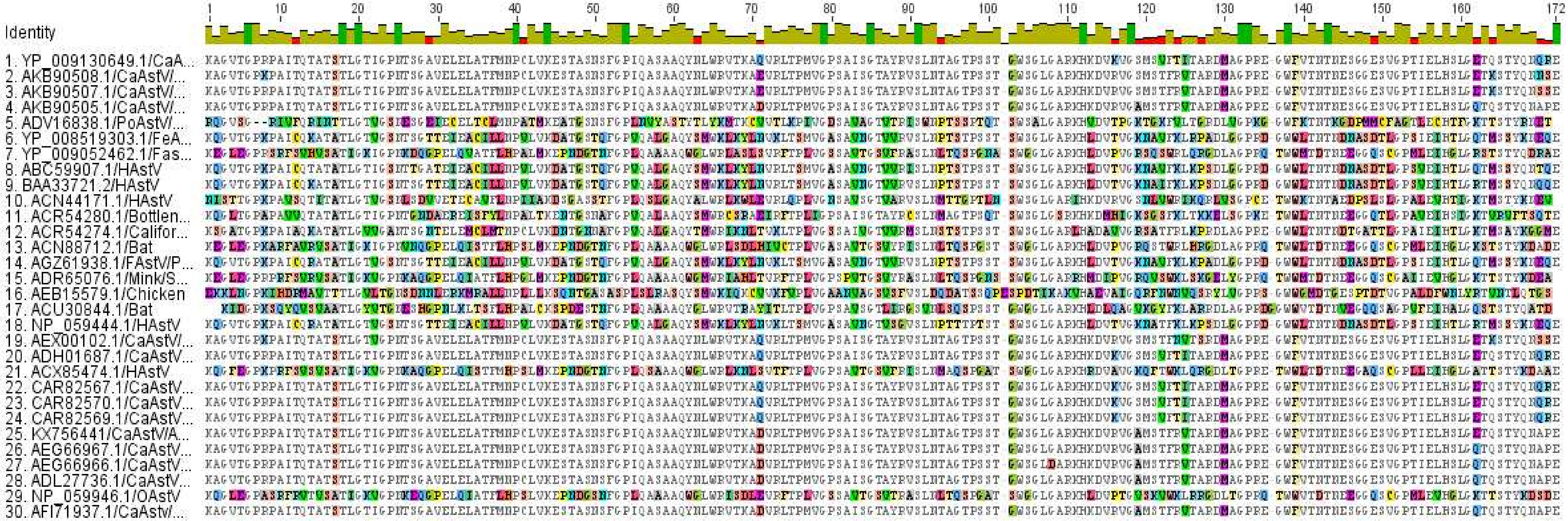

Figure S2: Multiple alignment of ORF2, conserved region. Disagreements to consensus sequence are highlighted.
